# Right-Sided Subcutaneous Implantable Cardioverter Defibrillator System Implantation in a Patient with Complex Congenital Heart Disease and Dextrocardia: A Case Report and Literature Review

**DOI:** 10.1155/2019/3907190

**Published:** 2019-02-05

**Authors:** Bandar Al-Ghamdi

**Affiliations:** ^1^Heart Centre, King Faisal Specialist Hospital and Research Centre (KFSH&RC), Riyadh, Saudi Arabia; ^2^Alfaisal University, Riyadh, Saudi Arabia

## Abstract

Patients with complex congenital heart disease (CHD) and low left ventricular ejection fraction are at an increased risk of sudden cardiac death (SCD). Prevention of SCD by subcutaneous implantable cardioverter defibrillator (S-ICD) implantation may represent a valuable option in certain CHD patients. Patients with CHD and dextrocardia pose a challenge in S-ICD system implantation, and nonstandard device placement may be required. Furthermore, electrocardiogram (ECG) screening prior to S-ICD implantation in CHD patients has significant limitations. This case represents the placement of a S-ICD system on the right side of the chest in a 26-year-old male with severe biventricular failure and nonsustained ventricular tachycardia following multiple corrective surgeries of situs inversus totalis, double-outlet right ventricle with a ventricular septal defect, and pulmonary atresia. The use of S-ICDs in a CHD population and in particular CHD patients with dextrocardia and right-sided S-ICD implantation is briefly discussed.

## 1. Introduction

Patients with complex congenital heart disease (CHD) and low left ventricular ejection fraction are at an increased risk of sudden cardiac death (SCD) [1–3]. Prevention of SCD by subcutaneous implantable cardioverter defibrillator (S-ICD) implantation may represent a valuable option in certain CHD patients. Patients with CHD and dextrocardia pose a challenge in S-ICD system implantation, and nonstandard device placement may be required.

The placement of a S-ICD system on the right side of the chest in a patient with complex CHD with dextrocardia and advanced heart failure is presented here.

The use of S-ICDs in a CHD population and in particular CHD patients with dextrocardia and right-sided S-ICD implantation is briefly discussed.

## 2. Case Presentation

**Figure 1:**
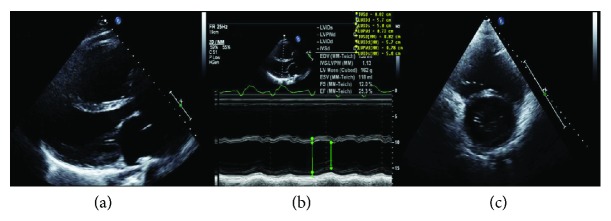
Echocardiogram: (a) parasternal long axis view showing right and left ventricular dilatation, (b) M-mode with left ventricular measurements, and (c) parasternal short axis view at mitral valve level with biventricular enlargement.

The patient is a 26-year-old male with a history of situs inversus totalis, double-outlet right ventricle with a ventricular septal defect, and pulmonary atresia, a type of tetralogy of Fallot (TOF). He underwent multiple corrective surgeries including biventricular repair in 1993 and tricuspid valve repair, residual ventricular septal defect (VSD) closure, and right ventricle (RV) to pulmonary artery (PA) homograft in 1997. Subsequently, he underwent a redo replacement of the pulmonary valve utilizing a cryopreserved pulmonary homograft with a size of 29 mm due to dysfunctional pulmonary homograft in 2010. A small residual ventricular septal defect with a restrictive left to right shunt (peak end -systolic gradient of 42 mmHg) and moderate tricuspid regurgitation with a peak gradient of 27 mmHg were noted in the echocardiogram. He had severe biventricular dysfunction (left ventricular ejection fraction < 25%, Figure 1) with frequent heart failure admissions requiring intermittent inotropic support, and he was on the waiting list for heart transplantation.

**Figure 2:**
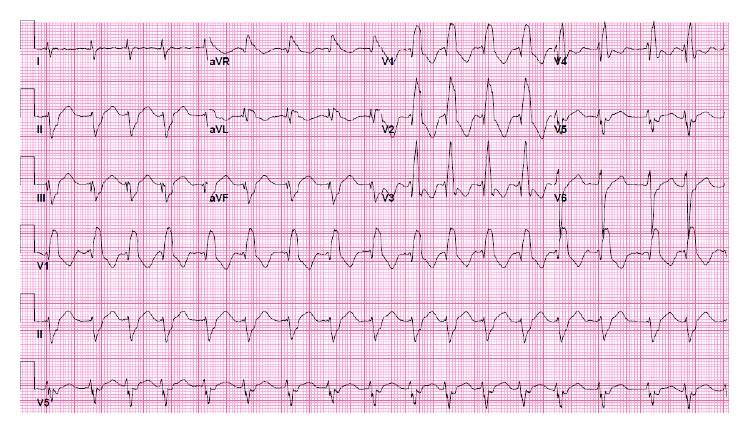
12-lead electrocardiogram showing atrial fibrillation with a ventricular rate of 104 beats per minute and right bundle branch block.

**Figure 3:**
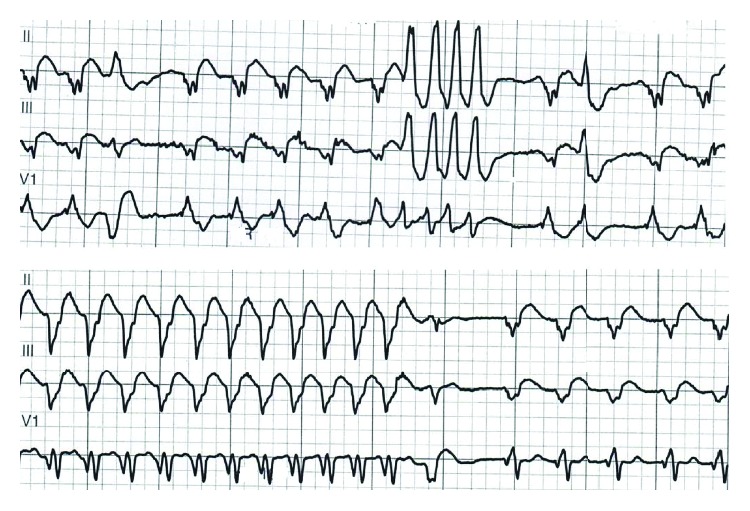
Nonsustained ventricular tachycardia on telemetry with two different morphologies.

The right heart catheterization showed low resistance (pulmonary vascular resistance index (PVRI) 1.3 Wood units (WU)), and shunt calculation showed a normal pulmonary flow (Qp) to systemic flow (Qs) ratio (Qp : Qs was 1 : 1). Both the inferior vena cava (IVC) and superior vena cava (SVC) were draining to the left-sided atrium. He also had intra-atrial reentry tachycardia with a ventricular rate of 117 beats per minute in 2012 and had external synchronized cardioversion once. He was considered for an electrophysiology study and ablation of the intra-atrial reentry tachycardia, but there was no significant change in his LVEF after cardioversion, and later on, he went into atrial fibrillation (AF) with a controlled ventricular rate. He was on anticoagulation with warfarin. An electrocardiogram (ECG) showed AF and right bundle branch block with a QRS duration of 164 milliseconds (ms) (Figure 2). He had premature ventricular complexes (PVCs) and runs of nonsustained ventricular tachycardia (VT) up to 5 beats at 187 beats per minute documented in telemetry and 24-hour Holter monitoring (Figure 3).

His other medical problems included acquired perforating dermatosis, folliculitis (hair follicle abscess), and bilateral lower limb varicose veins. His skin swab was positive for a methicillin-resistant *Staphylococcus aureus* (MRSA).

**Figure 4:**
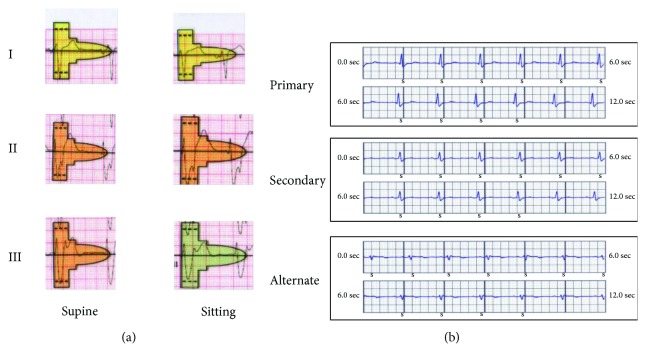
(a) Manual ECG screening test showing only lead I (alternate vector) in supine and sitting positions as acceptable vector at 10 mm/mV. (b) Postimplantation S-ECG sensing with gain setting 1X showing adequate sensing in the primary, secondary, and alternate vectors. The primary vector was automatically selected. S: subcutaneous.

The case was discussed in the cardiology meeting, and it was felt that he has a high risk of ventricular arrhythmias and SCD. It was also decided that S-ICD would be the best option for him considering his anatomy with residual VSD and a high risk of infection due to folliculitis and positive MRSA which may put him at risk of infective endocarditis with a transvenous implantable cardioverter defibrillator (TV-ICD). The S-ICD ECG screening showed only an alternate vector to be acceptable in the supine and sitting positions (Figure 4(a)).

The risks, benefits, and alternative of the procedure were all discussed with the patient including the risk of inappropriate ICD shocks. He agreed to the procedure, and informed consent was obtained.

The patient underwent S-ICD implantation (Emblem S-ICD (model A209) and S-ICD electrode (lead) (model 3401), Boston Scientific, Marlborough, MA) on the right side of the chest in December 2016. The pulse generator was placed at the right midaxillary line between the 5th and 6th intercostal spaces, and the S-ICD electrode was placed on the right parasternal area utilizing the standard intermuscular three-incision technique. Careful attention was made to avoid sternal wire contact with the S-ICD electrode.

**Figure 5:**
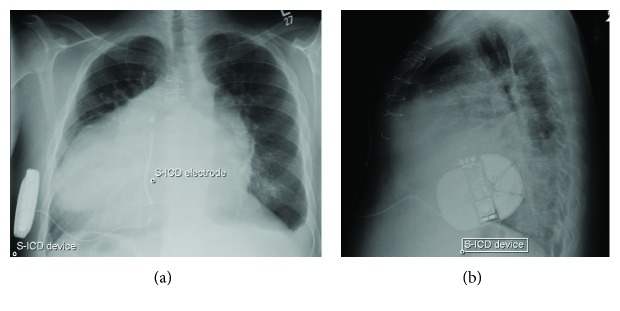
Posteroanterior (a) and lateral (b) chest x-rays showing situs inversus totalis, pectus excavatum, sternal wires from previous cardiac surgeries, and the S-ICD device and electrode. Note the presence of cardiomegaly, small left pleural effusion, and basilar atelectasis. S-ICD: subcutaneous implantable cardioverter defibrillator.

The S-ICD analysis at the end of the procedure revealed acceptable three sensing factors (primary, secondary, and alternate) (Figure 4(b)). Defibrillation threshold testing was not performed due to the concern of severe biventricular dysfunction. There was no T-wave oversensing with a limited exercise test on the first-day post device implantation. The chest X-ray showed an acceptable lead and device position (Figure 5). The patient made a good recovery with no complication related to the procedure.

During the follow-up period of 22 months, he had no sustained ventricular arrhythmia, and he did not have any appropriate or inappropriate ICD shocks.

## 3. Discussion

Implantation of a TV-ICD may be challenging or even impossible in patients with CHD due to complex cardiac and vascular anatomy. Furthermore, implantation of transvenous or epicardial systems in these patients is associated with short- and long-term risks. The incidence of venous occlusion, inappropriate shocks, and lead fractures is higher in CHD patients compared to non-CHD patients [4–6].

S-ICD represents an attractive alternative to TV-ICD in CHD patients. However, there is limited data on the S-ICD use in these patients' population. In the EFFORTLESS (Evaluation of Factors Affecting the Clinical Outcome and Cost-Effectiveness) registry, only thirty-three patients (7%) had CHD [7].

The analysis of the CHD cohort in the pooled data from the IDE (investigational device exemption) study and the EFFORTLESS registry including 19 out of 865 patients, after exclusion of patients with hypertrophic cardiomyopathy or cardiac channelopathies (Brugada syndrome, arrythmogenic right ventricular cardiomyopathy, and long QT syndrome), shows that S-ICD is a safe option in CHD patients at risk of SCD [8]. It has similar complication rates for the CHD versus non-CHD groups (10.5 vs. 9.6% [*p* = 0.89]) and similar rate of inappropriate shocks for both groups (10.5% vs. 10.9% [*p* = 0.96]). Successful defibrillation testing at 80 J was comparable for the two groups (100% in CHD vs. 98.5% in non-CHD, *p* = 0.62), but there was a significant difference found with threshold testing at 65 J with lower success in CHD patients (88.2% in CHD vs. 94.6% in non-CHD, *p* = 0.26) [8]. In another study that looked at the long-term experience with S-ICD in teenagers (<20 years of age) and young adults (20 to 26 years of age), thirty-one patients were included: thirteen were teenagers, and eighteen were young adults with a comparison to an age-matched control group with TV-ICDs. However, only four patients with CHD were included in this study. Ventricular arrhythmias were adequately terminated in eight patients (25.8%), and oversensing was observed in five patients (16.1%), resulting in at least one inappropriate shock. Younger age was an independent predictor of inappropriate shocks in S-ICD (hazard ratio: 0.56; 95% confidence interval: 0.34 to 0.92; *p* < 0.05) [9]. However, the rates of inappropriate shocks were comparable to those in patients with TV-ICDs [9].

Our patient has only one acceptable sensing vector with manual ECG screening. An automated screening tool was not available at the time of device implantation. However, during implantation procedure, all sensing vectors were acceptable. Patients with CHD commonly have conduction system disease with prolonged QRS duration which is a predictor of failed screening [10]. However, there were no significant differences observed in S-ICD eligibility between complex CHD patients and controls in a study that evaluated ECG vector screening in thirty patients with CHD and ten control subjects [11]. The alternate and primary vectors were most suitable in the complex CHD patients (tetralogy of Fallot (TOF), transposition of great arteries (TGA), Fontan circulation, and single ventricle physiology (SVP)). Furthermore, no significant impact of the postural change was observed for S-ICD eligibility compared to morphologically normal heart patients [11].

ECG screening may not be very accurate, and preprocedure screening with an external S-ICD to evaluate sensing at rest and during exercise in all three sensing vectors (algorithm-based screening) was shown in a small study to improve patients' selection and reduce the number of false-positive and false-negative ECG screening of the standard screening method [12]. There is no study with algorithm-based screening in CHD patients.

Effective implantation of S-ICD on the right side of the chest in patients with dextrocardia and CHD was described in two previous case reports [13, 14]. However, our patient has more advanced heart failure compared to the previously reported cases and on the heart transplant list, and we have about two years of follow-up with no problems.  Table 1 summarizes the reported cases with right-sided S-ICD and comparison to the current patient. Patients with CHD may require bradycardia or cardiac resynchronization therapy pacing. The S-ICD system can be used in conjunction with a transvenous pacing system if bradycardia pacing is needed [14].

Coordinating S-ICD with a leadless pacemaker is another novel approach that may convert arrhythmias with anti-tachycardia pacing (ATP) instead of a shock and provide bradycardia pacing at the same time. An early animal study with this approach is encouraging. However, studies in humans are still awaited [15, 16].

## 4. Conclusions

A S-ICD system is an attractive option for CHD patients with a risk of SCD and vascular access problems and intracardiac shunts at a high risk of device infection with a risk of bacteremia and infective endocarditis. In patients with dextrocardia, right-sided S-ICD implantation is feasible and effective.

## Figures and Tables

**Table 1:** Comparison of the reported cases with right-sided S-ICD implantation and current case.

Study year, author	Diagnoses	Surgeries/procedures	Reason to implant S-ICD	Device	Follow-up
2015, Waller et al. [13]	A 31-year-old female with TGA, situs inversus with dextrocardia Out-of-hospital VF arrest	Mustard procedure aged 18 months Transvenous dual chamber pacemaker in situ for bradycardia Atrial lead revision due to failure to capture of the original lead	Potential risk of baffle stenosis and superior vena cava obstruction with transvenous system	Boston Scientific SQ-RX S-ICD	—

2014, Ceresnak et al. [14]	A 21-year-old man with a history of dextrocardia, TOF, and Klinefelter syndrome	Multiple cardiac surgeries including placement of a right modified BT shunt in the newborn period, complete repair of TOF at age 1 year, PV replacement with intraoperative cryoablation of the RV/RVOT due to VT, TV annuloplasty, and modified RA maze procedure because of recurrent atrial flutter	Epicardial ICD system could be not implanted due to abdominal compartment syndrome and recurrent fever and the concern for infection	Boston Scientific SQ-RX S-ICD	—
	History of VT and easily inducible VF in the EPS.	Left-sided transvenous ICD at the of age 14 years with placement of a subcutaneous coil due to failed DFT Repeat PV replacement, TV replacement for severe TR and PR, RVOT patch augmentation, and partial removal of the transvenous ICD system			

Current case	A 26-year-old male situs inversus totalis, double-outlet RV with a VSD, and pulmonary atresia (a type of TOF). Severe biventricular dysfunction, on the waiting list for heart transplantation Nonsustained VT	Multiple corrective surgeries including biventricular repair in 1993 and TV repair, residual VSD closure, and RV to PA homograft in 1997 redo replacement of the PV utilizing a cryopreserved pulmonary homograft due to dysfunctional pulmonary homograft in 2010	A small residual VSD	Boston Scientific Emblem A209 S-ICD	22 months

BT: Blalock–Taussig; DFT: defibrillation threshold testing; EPS: electrophysiology study; ICD: implantable cardioverter defibrillator; PA: pulmonary artery; PR: pulmonary regurgitation; PV: pulmonary valve; RA: right atrial; RV: right ventricle; RVOT: right ventricular outflow tract; S-ICD: subcutaneous implantable cardioverter defibrillator; TOF: tetralogy of Fallot; TGA: transposition of the great arteries; TV: tricuspid valve; VF: ventricular fibrillation; VSD: ventricular septal defect; VT: ventricular.
